# Autoantibodies as potential prognostic factors for clinical outcomes related to COVID-19: a systematic review of inception prospective cohort studies with GRADE recommendations

**DOI:** 10.1590/1414-431X2024e13965

**Published:** 2025-01-31

**Authors:** F.C. Araújo, A.C.D. Amaral, H.J. Silva, J.N.V. Santos, V.A. Mendonça, V.C. de Oliveira, E. Rocha-Vieira

**Affiliations:** 1 Programa de Pós-Graduação em Ciências da Saúde, Laboratório de Biologia do Exercício e Imunometabolismo, Faculdade de Medicina, Universidade Federal dos Vales do Jequitinhonha e Mucuri, Diamantina, MG, Brasil; 2 Programa de Pós-graduação em Reabilitação e Desempenho Funcional, Laboratório de Inflamação e Metabolismo, Faculdade de Ciências Biológicas e da Saúde, Universidade Federal dos Vales do Jequitinhonha e Mucuri, Diamantina, MG, Brasil

**Keywords:** Systematic review, Autoantibodies, COVID-19, Anti-interferon antibodies, Prospective studies

## Abstract

This systematic review of inception prospective cohort studies aimed to investigate whether autoantibodies are potential prognostic factors for short- and long-term clinical outcomes of COVID-19. Searches were conducted in MEDLINE, EMBASE, AMED, GLOBAL HEALTH, and COCHRANE databases from 2019 to 2022. When possible, meta-analysis was conducted, otherwise findings from individual studies were reported using odds ratios (OR) with 95% confidence intervals (CI). Quality of evidence was summarized using the GRADE criteria. We identified 2292 references, 18 inception prospective cohort studies (3178 patients) were included in the systematic review, and 12 studies reached criteria for meta-analysis. Studies achieved, in general, low to moderate risk of bias. Moderate quality of evidence showed that anti-interferon (IFN) was associated with increased risk of severity (OR=7.75; CI=1.79-33.61) and mechanical ventilation (OR=4.19; CI=2.06-8.53), but not with COVID-19 mortality (OR=1.68; CI=0.63-4.44). Antiphospholipids were not associated with COVID-19 mortality (OR=1.42; CI=0.85-2.37; P=0.18; I^2^=3.21) nor with thrombosis risk (OR=1.41; CI: 0.71-2.8; P=0.33). Antinuclear antibody level was not associated with risk of mortality or severity (risk for mortality: OR=3.8; CI=0.78-18.6; P=0.1; I^2^: 32.3; severity: OR=1.74; CI=0.96-3.16; P=0.07). Evidence currently available is insufficient for a quantitative analysis of autoantibodies association with long COVID-19. Anti-IFN measurement should be considered in COVID-19 follow-up. In a population-based rational, optimized vaccination strategies should be considered for individuals with anti-IFN antibodies since it could represent a risk for a worse prognosis. High-quality prospective studies for short- and long-term disease effects and autoantibody evaluation are still needed.

## Introduction

The Coronavirus Disease-19 (COVID-19), caused by the severe acute respiratory syndrome coronavirus-2 (SARS-CoV2), leads to a variety of clinical conditions that comprise, in most cases, flu-like symptoms (1). However, other symptoms such as diarrhea, anosmia, dysgeusia, in addition to thrombotic events and viral/inflammatory pneumonia in more severe cases, can also occur (2,3). It has been proposed that hyperinflammation, commonly associated with other clinical conditions, including cytokine storm syndrome (CSS) and macrophage activation syndrome (MAS), is involved in the pathophysiological mechanism of severe COVID-19 (4,5). CSS and MAS are classically found in autoimmune diseases such as systemic lupus erythematosus, Still's disease, and catastrophic antiphospholipid antibody syndrome, among others (6-8). Because autoantibodies can play a major role in autoimmunity hyperinflammation and pathophysiology, they could also contribute to COVID-19 severity (9).

For instance, neutralizing autoantibodies against interferon (IFN)-omega and IFN-alfa (isolated or in combination) were found in 101 of 987 patients (10.2%) with life-threatening COVID-19 pneumonia at the onset of the critical phase (10). A correlation was also found between anti-DNA (OR=7.2, P=0.006) and anti-phosphatidylserine (PS) antibodies (OR=5.7, P=0.043) and disease severity after adjustment for age, race, and sex (11). Antiphospholipid and anti-*MDA5* (melanoma differentiation-associated gene 5) were detected in COVID-19 patients, and higher titers of these autoantibodies were associated with disease severity and unfavorable outcomes (intensive care, much longer disease course at discharge, and higher incidences of respiratory failure, shock, and other organ dysfunction) (12,13). Evidence of an association between autoantibodies and COVID-19 prognosis has practical clinical implications, given that different autoantibody types may be related to predicted clusters of symptoms or even to the individual's ability to control the infection (14). One mechanism for this triggering could be molecular mimicry (15). In addition, autoimmune phenocopies of inborn errors of cytokines, with neutralizing autoantibodies against cytokines are also well known (14), or in genetically predisposed patients the first neutrophil immune response may lead to antigen presentation and chronic immune dysregulation in small vessel vasculitis (9).

More recently, the term long COVID-19 was introduced, which relates to the long-term symptoms and clinical consequences of SARS-CoV-2 infection (16). Long COVID-19 is defined by the World Health Organization (WHO) as the continuation or development of new symptoms three months after the initial SARS-CoV-2 infection that cannot be explained by other causes (17). The cause of this post-viral syndrome is not known, but because patients with long COVID-19 present with prolonged multi-system involvement and significant disability, its association with autoantibodies has been proposed (16,18).

Identifing true prognostic factors of the disease is important as they can help to improve risk stratification, treatment, and lifestyle decisions, and the design of randomised trials. Prognostic factor studies can sometimes be of variable quality with inconsistent findings (19,20). This systematic review of inception prospective cohort studies aimed to identify the association between autoantibodies and prognosis in COVID-19. It is hypothesized that autoantibodies increase the risk of disease severity and the occurrence of long COVID-19.

## Methods

This review was conducted following the Preferred Reporting Items for Systematic Reviews and Meta-Analysis (PRISMA) guidelines (21), and it is registered in the International Prospective Register of Systematic Reviews (PROSPERO - CRD42022342477).

### Research question

The proposal of this systematic review was to answer the following question: Are autoantibodies (and which ones) COVID-19 outcome predictors? A modified PICOT strategy (19) was used to define the research question components: Participants (P) = COVID-19 patients; Index/list of prognostic factors (I) = autoantibodies presence; Outcome (O) = Death, autoimmune diseases, disease severity, pulmonary involvement, chronic pain, joint pain, fatigue, long COVID-19, thrombotic events, hospital stay length, intensive care need, invasive and non-invasive mechanical ventilation need, use of vasopressors; T (Time) = autoantibodies evaluated within 30 days of illness (1) and after a month from illness onset (2), with the cut-off point based on infectivity period (22). Because this is a prognostic factor review, comparators (C) were not included.

### Inclusion and exclusion criteria

Inclusion criteria were articles reporting original data on autoantibodies in adult (>18 years) COVID-19 patients diagnosed necessarily by RT-PCR performed within 30 days of the onset of symptoms (22,23). Only longitudinal and prospective cohort studies were included. Duplicate data, qualitative studies, case reports, case series, conference reports and comments, editorials, and expert opinions were excluded.

### Search strategy

Articles were searched up to July 4, 2022 in the following databases: MEDLINE/PUBMED, EMBASE, AMED, GLOBAL HEALTH, and COCHRANE, using the Ovid Interface (Ovid Technologies, Inc., https://www.wolterskluwer.com/en/solutions/ovid). There was no language or date restriction. We also searched for articles in the references of systematic reviews (hand searching). Descriptors were searched at https://www.ncbi.nlm.nih.gov/mesh/. Terms for prospective studies (study design), COVID-19 (population), and autoantibodies (prognostic factors) were chosen. No specific terms related to the outcomes of interest were used to increase search sensitivity and avoid missing possibly relevant studies and other relevant outcomes. The search strategy and criteria employed are shown in Supplementary Figure S1.

### Study selection and data extraction

All publications found on databases were exported to Endnote and duplicates were removed. Then, two independent reviewers (F.C.A. and A.C.D.A.) screened titles and abstracts and assessed the full texts for eligibility criteria. Disagreements were solved by a third reviewer (J.N.V.S.).

Extracted data included author names, date of publication, type of study, city, country, sample source, sample size, patient comorbidities, study design-inception cohort, diagnostic tests, clinical presentation details, blood tests, radiological findings, treatment therapies description, hospitalization and intensive care unit (ICU) admission, and clinical course (death, complications, readmission, discharge). When data were not presented in a usable way, authors were contacted, with up to three attempts, with two days in between.

According to the CHARMS-PF (Checklist for Critical Appraisal and Data Extraction for Systematic Reviews of prognostic factor studies) tool (19), the following data were extracted from selected articles: age (or mean age), sex or male/female ratio, the method for COVID-19 diagnosis, length of hospital stay (if applicable), disease severity indicators (need for mechanical ventilation, intensive care, vasopressors, oxygen therapy, occurrence of thrombotic events), previous comorbidities, place of origin, antibodies evaluated (types and measurement techniques), collection time, previous medications, mortality rate, sample size, risk estimate defined by odds ratio (for binary outcomes) or hazard ratio (for time-related outcomes), difference in means (for ongoing outcomes), and logistic regression performance.

### Risk of bias evaluation

Two independent reviewers (F.C.A. and A.C.D.A.) assessed methodological quality of included studies using the Quality in Prognosis Studies (QUIPS) tool (19). The tool assesses six domains: i) study participation; ii) study attrition; iii) prognostic factor measurement; iv) outcome measurement; v) study confounding, and vi) statistical analysis and reporting. Each domain was rated as having high, moderate, or low risk of bias. Disagreements were solved by a third reviewer (J.N.V.S.). Reviewers used a standardized form downloaded from the Cochrane Methods Prognosis website and were previously trained on how to use the form.

### Quantitative synthesis (meta-analysis)

After data extraction and risk of bias assessment, meta-analysis was performed when feasible, using the Comprehensive Meta-analysis program, version 3.0 (Biostat, USA). For summarizing results, number of patients, means and standard deviations, differences in means (when possible), or difference in standard deviations were used. We also considered odds ratio and hazard ratio measurements. Weights were assigned to each article evaluated. A 95% confidence interval and P<0.05 were used for significant differences. Data were presented as Forest plots. Heterogeneity (I^2^) was estimated (moderate heterogeneity was defined as I^2^ >50%). Random effects model was used. Effect size was estimated by odds ratio percentage.

Subgroup analyzes for specific types of autoantibodies assessment were conducted and meta-analysis perfomed when possible, considering differences in means, standard deviations, and risk measures, including their relationships with certain outcomes.

### Assessment of the quality of evidence

The quality level of the evidence was defined as high, moderate, low, or very low using GRADE (Grading of Recommendations, Assessment, Development and Evaluations) domain, after meta-analysis. The quality of the evidence of the reviews started at high and was downgraded by one level based on five criteria: indirect evidence; inconsistency (when I^2^ was higher than 50% or when pooling was not possible (24)); imprecision (25); publication bias; and methodological limitations. When 25% or more studies were highly biased, quality of evidence was downgraded by 2 levels (26). High magnitude effect was considered a criterion to raise evidence by one level (27).

## Results

**Figure 1:**
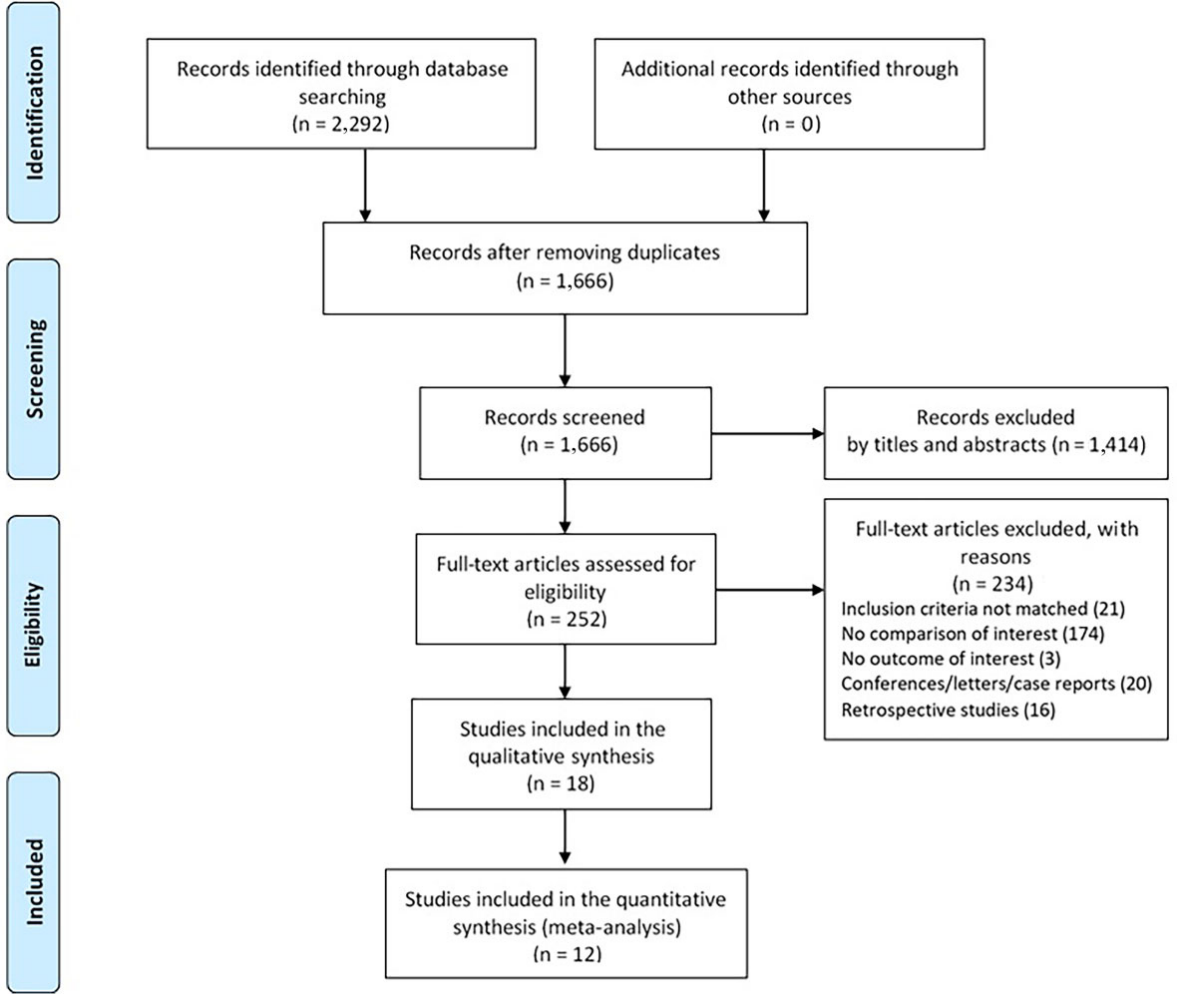
Flow of studies evaluated through the review process.

A total of 2,292 articles were obtained and, after duplicate deletion, 1,666 studies were evaluated by the title and abstract (Figure 1). A total of 252 articles were selected for full reading, of which 71 were eligible for data extraction. Of these, 18 studies remained for qualitative and methodological analysis (Supplementary Table S1 (9,28-44)). Twelve studies reached criteria for quantitative synthesis (meta-analysis). The assessment results of methodological quality are shown in Table 1 (9,28-44). None of the studies presented full low risk of bias.

**Table 1:** Methodological assessment of the studies by Quality in Prognostic Studies (QUIPS).

Authors	Study participation	Study attrition	Prognostic factor measurement	Outcome measurement	Study confounding	Statistical analysis and reporting
Abers et al. 28	moderate	low	low	low	moderate	low
Gonen et al. 29	low	low	low	moderate	moderate	moderate
Guasp et al. 30	moderate	high	low	moderate	moderate	high
Karahan et al. 31	moderate	moderate	low	low	high	moderate
L'Huillier et al. 32	moderate	low	low	low	moderate	low
Lui et al. 33	low	low	low	low	high	high
Najim et al. 34	low	low	low	low	low	low
Pagano et al. 35	moderate	moderate	low	low	high	high
Pascolini et al. 9	low	low	low	low	high	high
Peker et al. 36	moderate	low	low	high	high	moderate
Petrikov et al. 37	moderate	low	low	moderate	moderate	moderate
Raadsen et al. 38	moderate	low	low	low	moderate	low
Rodriguez-Perez et al. 39	moderate	low	low	low	moderate	high
Sacchi et al. 40	low	low	low	moderate	moderate	high
Seeble et al. 41	moderate	moderate	low	low	moderate	moderate
Serrano et al. 42	low	low	low	low	moderate	low
Su et al. 43	moderate	low	low	low	moderate	low
Taeschler et al. 44	moderate	low	low	moderate	moderate	low

Several autoantibodies, at least 40 types in all articles searched, were studied for associations with COVID-19 outcomes. In the same way, several outcomes (clinical and laboratory) have been described. However, for this review, we studied only clinical outcomes and antibodies derived from prospective studies.

In total, there were 2,215 COVID-19 patients (outpatients and inpatients) from several countries and continents (Italy, Turkey, Spain, China, United States, and Russia, for instance). All patients were ≥18 years old and had a confirmed COVID-19 diagnosis by RT-PCR. Data used in the meta-analysis were obtained for patients evaluated between February 2020 and May 2021. None of these patients was cited as vaccinated.

We extracted quantitative data from the following autoantibodies: anti-interferon (anti-IFN), anti-pituitary, anti-hypothalamus, antineuronial, antiphospholipids, anti-ACE 2 (angiotensin converting enzyme 2), anti-angiotensin I, antithyroid, anti-apoliprotein A1, antinuclear antibody (ANA) factor and derivatives, and anticytoplasmic and neutrophilic antibodies (ANCA). Data for the other antibodies were not evaluated because they came from non-prospective studies.

**Figure 2:**
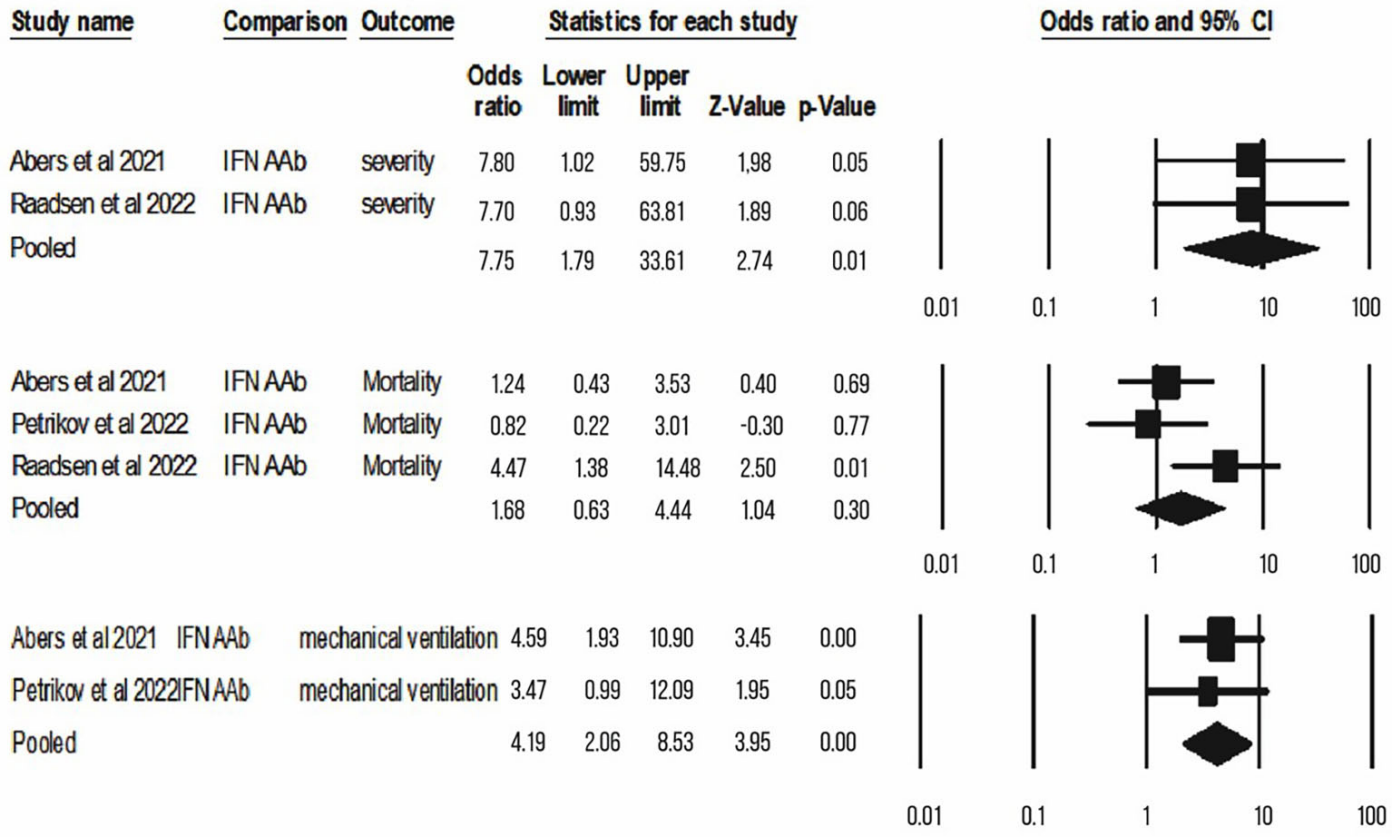
Summary of the evidence of the association between anti-IFN and COVID-19 outcomes of mortality, severity, and need for mechanical ventilation (Forest plot). IFN A Ab: IFN-alpha antibody.

**Figure 3:**
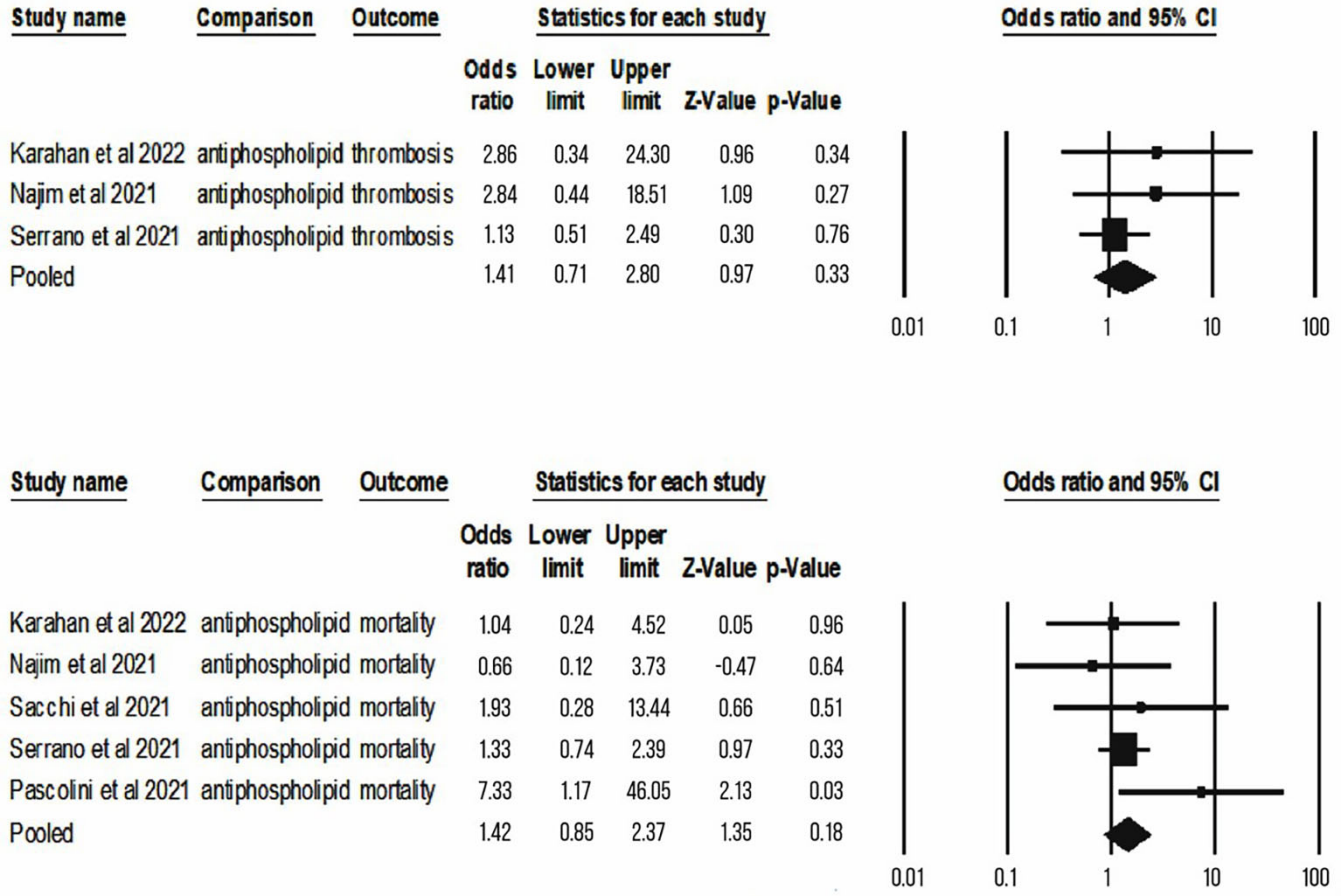
Summary of the evidence of the association between antiphospholipids and COVID-19 outcomes of mortality and thrombosis (Forest plot).

**Figure 4:**
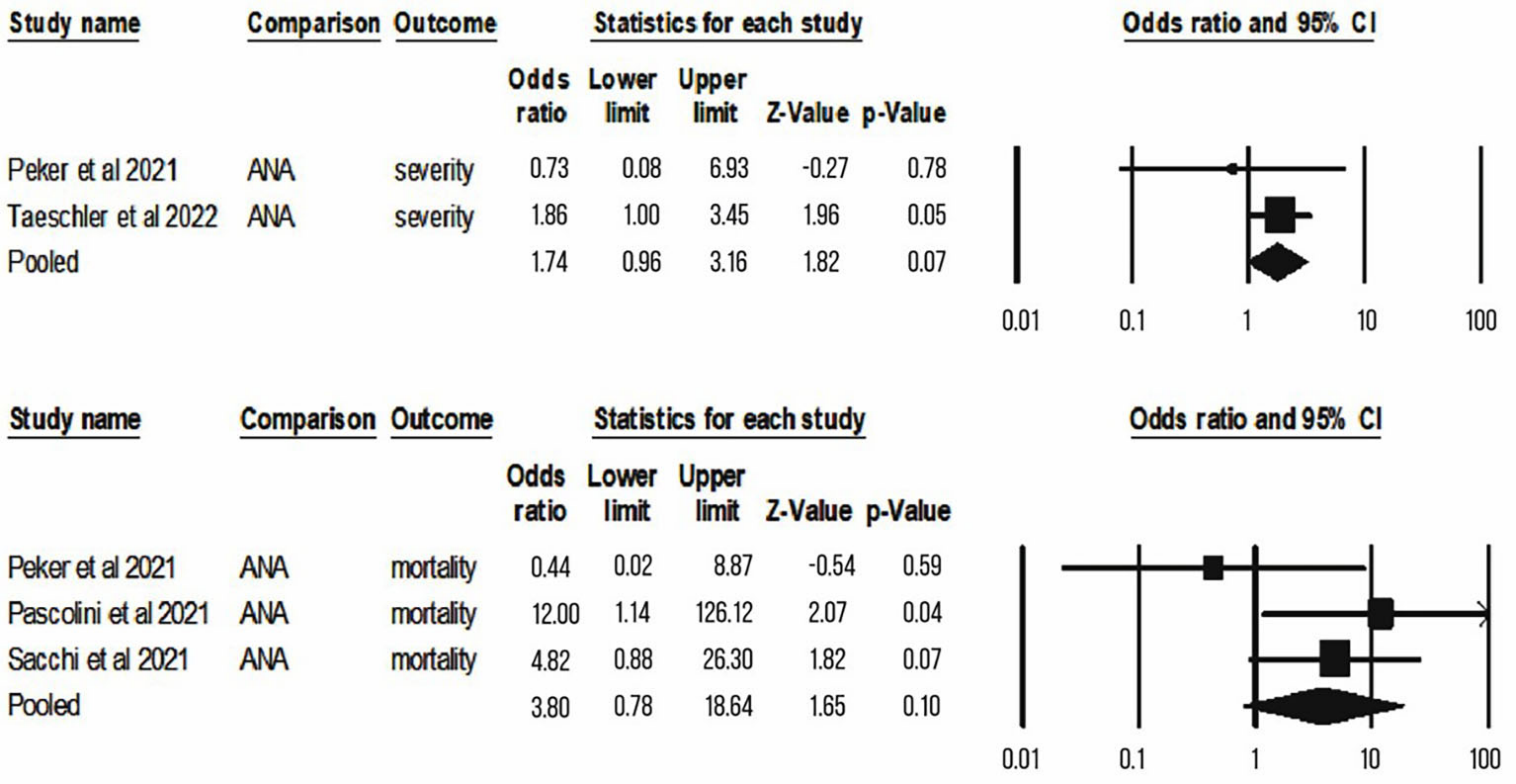
Summary of the evidence of the association between antinuclear antibodies and COVID-19 outcomes of mortality and severity. ANA: antinuclear antibodies.

For quantitative synthesis, we obtained comparable data (at least 2 studies with similar metrics and outcomes) for anti-interferon, antiphospholipids, and ANA, as follows. The presence of anti-IFN increased the risk of severe disease (OR=7.75; CI=1.79-33.61; P=0.01) and mechanical ventilation in patients with COVID-19 (OR=4.19; CI=2.06-8.53; P<0.001), but not mortality (OR=1.68; CI=0.63-4.44; P=0.3; I^2^=51.8) (Figure 2). Antiphospholipid antibodies did not increase the risk of mortality (OR=1.42; CI=0.85-2.37; P=0.18; I^2^=3.21) and thrombosis (OR=1.41; CI=0.71-2 .8; P=0.33) (Figure 3). A similar result was observed for antinuclear antibodies, with no association with risk of mortality (Figure 4) (OR=3.8; CI=0.78-18.6; P=0.1; I^2^=32.3) and COVID-19 severity (OR=1.74; CI=0.96-3,16; P=0.07).

We also found studies that decribed the association of other autoantibodies and COVID-19 outcomes, however, in an insuffcient number for meta-analysis. Levels of anti-ACE 2 and anti-angiotensin I, for example, were correlated with increased risk for COVID-19 (39). Also, the risk for long COVID-19 was increased in patients positive for ANA (41). The percentage of women with body aches with ANA titers >1/160 after 12 months of COVID-19 was 46.7 *vs* 8.7% between women with no or lower ANA titers (P=0.003). Also, there was an association between higher ANA titers and the frequency of neurological symptoms (66.7 *vs* 26.10%, P=0.03). Additionally, the presence of ANCA was associated with a higher risk of COVID-19 mortality (40 *vs* 23%; OR=2.19; CI=0.4781-10.03; P=0.31) (40) and severe disease (19.7 *vs* 3.6%; OR=6.25; CI=1.449-26.9; P=0.01) (44).

According to GRADE, the level of evidence for an association between autoantibodies and COVID-19 prognosis is very low, as most studies had at least one association with heterogeneity greater than 50%, and high methodological bias was found in more than 25% of the studies. However, the level of evidence for findings related to anti-IFN was considered moderate.

## Discussion

This is the first systematic review to assess the association between the prognosis of COVID-19 and the highest possible number of autoantibodies. Our results draw attention mainly to the role of anti-IFN antibodies in COVID-19. We also found data suggesting the involvement of other autoantibodies (antiphospholipids, ANA) in COVID-19 prognosis.

We found an increased risk of severe COVID-19 with the presence of anti-IFN antibodies, as they probably facilitate virus evasion mechanisms (45). SARS-CoV-2 inhibits the triggering of innate immunity (type I IFN, for instance), which is responsible, among other functions, for triggering the antiviral state, delaying viral spread until more specific and specialized mechanisms of the adaptive immunity are activated (45). Severe or fatal COVID-19 is largely due to lung immunopathology (neutrophilic inflammation) triggered by high viral loads and a lack of timely T cell responses (45,46). This model can explain why anti-IFN antibodies are associated with COVID-19 severity, as it is an important cytokine for virus replication and spread control (47). Neutralization of IFN by autoantibodies can hinder initial viral control and thus increase the chance of viral spread and exacerbated inflammatory response, leading to more severe manifestations and, consequently, the need for more aggressive interventions such as mechanical ventilation. Of note, we found that anti-IFN was also associated with increased risk for mechanical ventilation in COVID-19 patients.

Immune mechanisms other than type I IFN contribute to virus elimination and viral infection termination. This can explain why anti-IFN was related to disease severity but not mortality risk, as reported by others (38). Also, while a lack of type I IFN signaling during early infection is detrimental for SARS-CoV-2 containment, type I IFN may contribute to secondary inflammation during the late hyperinflammatory stage of COVID-19 (28). Furthermore, while IFN administration before the viral peak and the inflammatory phase of the disease may have a strong protective effect, IFN treatment during the inflammatory and severe stages causes long-lasting harm to patients (48). Therefore, the reduced type I IFN triggered by anti-IFN autoantibodies in advanced stages of the disease (not during early infection) can explain the lack of association with mortality risk.

Impairment of IFN activity can exacerbate the inflammatory responses in severe COVID-19 patients (49). An aberrant adaptive immune response against SARS-COV-2 can induce autoantibody formation (50,51). Occurrence of antiphospholipid antibodies, commonly found in antiphospholipid syndrome (a disease in which thrombotic events are the main clinical manifestations), and ANA, commonly found in systemic lupus erithematosus, in COVID-19 may be related to the severe COVID-19 physiopathology, marked by neutrophil activation and NETosis in the acute phase of the disease (52,53).

So far, it is not possible to establish an association between other autoantibodies reported in COVID-19 patients and disease outcomes, including antiphospholipids and ANA, as proposed by others (12,54). Antiphospholipids can contribute to COVID-19 thrombotic events, leading to neutrophil activation and liberation of extracellular traps and initiating thromboinflammation at the acute phase of the disease (52,53). Although Pascolini et al. (9) found increased COVID-19 mortality among patients who tested positive for ANA and antiphospholipids, none of the prospective studies included in this review reported a significant association between antiphospholipids and thrombosis risk. Thus, the clinical significance of these autoantibodies in COVID-19 patients remains to be determined.

In addition, the currently available evidence is insufficient for a quantitative analysis (meta-analysis) of autoantibodies association with long COVID-19. However, Seeble et al. (41) reported a correlation between ANA titers and long COVID-19, including neurocognitive symptoms and dyspnea, and speculated a potential contribution of autoimmunity for the persistence of symptons. It can be hypothesized that IFN neutralization and exacerbated inflammatory response in severe COVID-19 patients could trigger autoimmunity (49) and increase the risk for long COVID-19. However, studies to support this association are still lacking.

It must be noted that the evidence presented here is based on COVID-19 data obtained in a pre-vaccine context. While this reduces the number of confounding factors (vaccination itself could trigger immunological manifestations (55)), it does not portray the current configuration of COVID-19. However, it is a model for the long-term consequences of the disease, as these patients will be the first to show possible long-term manifestations of the disease. In addition, it is currently unclear whether autoantibodies can affect the response to vaccines and whether a vaccine-triggered immune response (or multiple doses) can overcome type I IFN deficiency in response to viral inocula. Vaccination strategies may vary depending on the individual's degree of immunosuppression, as is the case with vaccination against hepatitis B (56). More studies are needed to clarify this issue in relation to COVID-19, anti-interferon antibodies, and vaccination.

## Conclusions

This review demonstrated that the risk of poorer outcomes (severity) in COVID-19 seems to be associated with the presence of anti-IFN antibodies, based on the moderate level of evidence. Anti-IFN measurement should be considered in the follow-up of COVID-19 patients, and it is worth discussing optimized vaccination strategies for individuals that present anti-IFNN antibodies. The lack of well-designed prospective studies was the main limitation of our analysis. Prospective studies evaluating long-term disease effects and autoantibodies are also still needed.

## Supplementary Material

Click to view [pdf].
