# Supplementary material for: Autoantibodies as potential prognostic factors for clinical outcomes related to COVID-19: a systematic review of inception prospective cohort studies with GRADE recommendations
Source: Braz J Med Biol Res. 2025 Jan 31;58:e13965. doi: 10.1590/1414-431X2024e13965 (PMC11793148; doi:10.1590/1414-431X2024e13965)
Supplement: Supplementary file 1 [file 1414-431X-bjmbr-58-e13965-suppl.pdf]

Figure S1. Detailed search strategy conducted on July 4, 2022. The bibliographic search was conducted using the OVID interface (Ovid Technologies, Inc), which provides access to online bibliographic databases in health sciences (Medline, Embase, Amed, Global Health). All the terms used are indicated (lines 1 to 78), as well as where in which paper the terms were searched (ab: abstract; hw: heading word; kw: keyword; ti: title; ot: original title; tn: trade name; dm: device manufacturer; mf: manufacturer; dv: device; kf: keyword heading; fx: subheading; dq: date qualification; cw: chemical words; nm: name of substance; ox: CAS registry number; px: pharmacologic action; rx: pharmacologic substances; ui: unique identifier; sy: systematic review; sh: subject heading; tx: full text; ct: clinical trial). How the searched terms were combined (lines 15, 26, 28 and 80) and the time limit (yr: year) (line 81) are also indicated.

OVID (MEDLINE, EMBASE, COCHRANE, AMED, GLOBAL HEALTH)

1. Longitudinal stud*.mp. [mp=ab, hw, ti, tn, ot, dm, mf, dv, kf, fx, dq, cw, nm, ox, px, rx, ui, sy, sh, kw, tx, ct]

2. Prospective Stud*.mp. [mp=ab, hw, ti, tn, ot, dm, mf, dv, kf, fx, dq, cw, nm, ox, px, rx, ui, sy, sh, kw, tx, ct]

3. Follow-up stud*.mp. [mp=ab, hw, ti, tn, ot, dm, mf, dv, kf, fx, dq, cw, nm, ox, px, rx, ui, sy, sh, kw, tx, ct]

4. Prognosis.mp. [mp=ab, hw, ti, tn, ot, dm, mf, dv, kf, fx, dq, cw, nm, ox, px, rx, ui, sy, sh, kw, tx, ct]

5. Survival.mp. [mp=ab, hw, ti, tn, ot, dm, mf, dv, kf, fx, dq, cw, nm, ox, px, rx, ui, sy, sh, kw, tx, ct]

6. Survival analysis.mp. [mp=ab, hw, ti, tn, ot, dm, mf, dv, kf, fx, dq, cw, nm, ox, px, rx, ui, sy, sh, kw, tx, ct]

7. Logistic model*.mp. [mp=ab, hw, ti, tn, ot, dm, mf, dv, kf, fx, dq, cw, nm, ox, px, rx, ui, sy, sh, kw, tx, ct]

8. Proportional hazards model*.mp. [mp=ab, hw, ti, tn, ot, dm, mf, dv, kf, fx, dq, cw, nm, ox, px, rx, ui, sy, sh, kw, tx, ct]

9. Disease Progression.mp. [mp=ab, hw, ti, tn, ot, dm, mf, dv, kf, fx, dq, cw, nm, ox, px, rx, ui, sy, sh, kw, tx, ct]

10. Life Table*.mp. [mp=ab, hw, ti, tn, ot, dm, mf, dv, kf, fx, dq, cw, nm, ox, px, rx, ui, sy, sh, kw, tx, ct]

11. cohort stud*.mp. [mp=ab, hw, ti, tn, ot, dm, mf, dv, kf, fx, dq, cw, nm, ox, px, rx, ui, sy, sh, kw, tx, ct]

12. prognos*.mp. [mp=ab, hw, ti, tn, ot, dm, mf, dv, kf, fx, dq, cw, nm, ox, px, rx, ui, sy, sh, kw, tx, ct]

13. course.mp. [mp=ab, hw, ti, tn, ot, dm, mf, dv, kf, fx, dq, cw, nm, ox, px, rx, ui, sy, sh, kw, tx, ct]

14. inception.mp. [mp=ab, hw, ti, tn, ot, dm, mf, dv, kf, fx, dq, cw, nm, ox, px, rx, ui, sy, sh, kw, tx, ct]

15. 1 or 2 or 3 or 4 or 5 or 6 or 7 or 8 or 9 or 10 or 11 or 12 or 13 or 14

16. COVID-19.mp. [mp=ab, hw, ti, tn, ot, dm, mf, dv, kf, fx, dq, cw, nm, ox, px, rx, ui, sy, sh, kw, tx, ct]

17. Coronavirus disease.mp. [mp=ab, hw, ti, tn, ot, dm, mf, dv, kf, fx, dq, cw, nm, ox, px, rx, ui, sy, sh, kw, tx, ct]

18. nCov.mp. [mp=ab, hw, ti, tn, ot, dm, mf, dv, kf, fx, dq, cw, nm, ox, px, rx, ui, sy, sh, kw, tx, ct]

19. 19nCov.mp. [mp=ab, hw, ti, tn, ot, dm, mf, dv, kf, fx, dq, cw, nm, ox, px, rx, ui, sy, sh, kw, tx, ct]

20. novel coronavirus.mp. [mp=ab, hw, ti, tn, ot, dm, mf, dv, kf, fx, dq, cw, nm, ox, px, rx, ui, sy, sh, kw, tx, ct]

21. novel coronavirus 2019.mp. [mp=ab, hw, ti, tn, ot, dm, mf, dv, kf, fx, dq, cw, nm, ox, px, rx, ui, sy, sh, kw, tx, ct]

22. Whuan coronavirus.mp. [mp=ab, hw, ti, tn, ot, dm, mf, dv, kf, fx, dq, cw, nm, ox, px, rx, ui, sy, sh, kw, tx, ct]

23. Whuan pneumonia.mp. [mp=ab, hw, ti, tn, ot, dm, mf, dv, kf, fx, dq, cw, nm, ox, px, rx, ui, sy, sh, kw, tx, ct]

24. Sars-CoV-2.mp. [mp=ab, hw, ti, tn, ot, dm, mf, dv, kf, fx, dq, cw, nm, ox, px, rx, ui, sy, sh, kw, tx, ct]

25. Severe acute respiratory syndrome.mp. [mp=ab, hw, ti, tn, ot, dm, mf, dv, kf, fx, dq, cw, nm, ox, px, rx, ui, sy, sh, kw, tx, ct]

26. 16 or 17 or 18 or 19 or 20 or 21 or 22 or 23 or 24 or 25

27. incidence stud*.mp. [mp=ab, hw, kw, ti, ot, tn, dm, mf, dv, kf, fx, dq, cw, nm, ox, px, rx, an, ui, sy, sh, tx, ct]

28. 15 or 27

29. Autoantibod*.mp. [mp=ab, hw, kw, ti, ot, tn, dm, mf, dv, kf, fx, dq, cw, nm, ox, px, rx, an, ui, sy, sh, tx, ct]

30. Anticardiolipin Antibod*.mp. [mp=ab, hw, kw, ti, ot, tn, dm, mf, dv, kf, fx, dq, cw, nm, ox, px, rx, an, ui, sy, sh, tx, ct]

31. Anticardiolipin.mp. [mp=ab, hw, kw, ti, ot, tn, dm, mf, dv, kf, fx, dq, cw, nm, ox, px, rx, an, ui, sy, sh, tx, ct]

32. Anti- Beta 2 Glycoprotein I antibod*.mp. [mp=ab, hw, kw, ti, ot, tn, dm, mf, dv, kf, fx, dq, cw, nm, ox, px, rx, an, ui, sy, sh, tx, ct]

33. Anti- Beta 2 Glycoprotein I.mp. [mp=ab, hw, kw, ti, ot, tn, dm, mf, dv, kf, fx, dq, cw, nm, ox, px, rx, an, ui, sy, sh, tx, ct]

34. Anti-beta2GPI-IgG.mp. [mp=ab, hw, kw, ti, ot, tn, dm, mf, dv, kf, fx, dq, cw, nm, ox, px, rx, an, ui, sy, sh, tx, ct]

35. Anti-beta2GPI-IgM.mp. [mp=ab, hw, kw, ti, ot, tn, dm, mf, dv, kf, fx, dq, cw, nm, ox, px, rx, an, ui, sy, sh, tx, ct]

36. Anti-beta-2 glycoprotein I.mp. [mp=ab, hw, kw, ti, ot, tn, dm, mf, dv, kf, fx, dq, cw, nm, ox, px, rx, an, ui, sy, sh, tx, ct]

37. Anti-B2GPI.mp. [mp=ab, hw, kw, ti, ot, tn, dm, mf, dv, kf, fx, dq, cw, nm, ox, px, rx, an, ui, sy, sh, tx, ct]

38. Anti beta-2-glycoprotein-I.mp. [mp=ab, hw, kw, ti, ot, tn, dm, mf, dv, kf, fx, dq, cw, nm, ox, px, rx, an, ui, sy, sh, tx, ct]

39. Anticardiolipin Cofactor*.mp. [mp=ab, hw, kw, ti, ot, tn, dm, mf, dv, kf, fx, dq, cw, nm, ox, px, rx, an, ui, sy, sh, tx, ct]

40. Anti-Neutrophil Cytoplasmic Antibod*.mp. [mp=ab, hw, kw, ti, ot, tn, dm, mf, dv, kf, fx, dq, cw, nm, ox, px, rx, an, ui, sy, sh, tx, ct]

41. Anti-Neutrophil Cytoplasmic.mp. [mp=ab, hw, kw, ti, ot, tn, dm, mf, dv, kf, fx, dq, cw, nm, ox, px, rx, an, ui, sy, sh, tx, ct]

42. Anti Neutrophil Cytoplasmic.mp. [mp=ab, hw, kw, ti, ot, tn, dm, mf, dv, kf, fx, dq, cw, nm, ox, px, rx, an, ui, sy, sh, tx, ct]

43. Anti-Neutrophil.mp. [mp=ab, hw, kw, ti, ot, tn, dm, mf, dv, kf, fx, dq, cw, nm, ox, px, rx, an, ui, sy, sh, tx, ct]

44. ANCA.mp. [mp=ab, hw, kw, ti, ot, tn, dm, mf, dv, kf, fx, dq, cw, nm, ox, px, rx, an, ui, sy, sh, tx, ct]

45. Antineutrophil Cytoplasmic.mp. [mp=ab, hw, kw, ti, ot, tn, dm, mf, dv, kf, fx, dq, cw, nm, ox, px, rx, an, ui, sy, sh, tx, ct]

46. Antineutrophil.mp. [mp=ab, hw, kw, ti, ot, tn, dm, mf, dv, kf, fx, dq, cw, nm, ox, px, rx, an, ui, sy, sh, tx, ct]

47. c-ANCA.mp. [mp=ab, hw, kw, ti, ot, tn, dm, mf, dv, kf, fx, dq, cw, nm, ox, px, rx, an, ui, sy, sh, tx, ct]

48. c ANCA.mp. [mp=ab, hw, kw, ti, ot, tn, dm, mf, dv, kf, fx, dq, cw, nm, ox, px, rx, an, ui, sy, sh, tx, ct]

49. p-ANCA.mp. [mp=ab, hw, kw, ti, ot, tn, dm, mf, dv, kf, fx, dq, cw, nm, ox, px, rx, an, ui, sy, sh, tx, ct]

50. p ANCA.mp. [mp=ab, hw, kw, ti, ot, tn, dm, mf, dv, kf, fx, dq, cw, nm, ox, px, rx, an, ui, sy, sh, tx, ct]

51. ACE2 autoantibod*.mp. [mp=ab, hw, kw, ti, ot, tn, dm, mf, dv, kf, fx, dq, cw, nm, ox, px, rx, an, ui, sy, sh, tx, ct]

52. Angiotensin-converting enzyme 2 autoantibod*.mp. [mp=ab, hw, kw, ti, ot, tn, dm, mf, dv, kf, fx, dq, cw, nm, ox, px, rx, an, ui, sy, sh, tx, ct]

53. Anti-interferon antibod*.mp. [mp=ab, hw, kw, ti, ot, tn, dm, mf, dv, kf, fx, dq, cw, nm, ox, px, rx, an, ui, sy, sh, tx, ct]

54. Antinuclear Factor*.mp. [mp=ab, hw, kw, ti, ot, tn, dm, mf, dv, kf, fx, dq, cw, nm, ox, px, rx, an, ui, sy, sh, tx, ct]

55. Antinuclear.mp. [mp=ab, hw, kw, ti, ot, tn, dm, mf, dv, kf, fx, dq, cw, nm, ox, px, rx, an, ui, sy, sh, tx, ct]

56. Antinuclear Antibod*.mp. [mp=ab, hw, kw, ti, ot, tn, dm, mf, dv, kf, fx, dq, cw, nm, ox, px, rx, an, ui, sy, sh, tx, ct]

57. Fluorescent Antinuclear Antibod*.mp. [mp=ab, hw, kw, ti, ot, tn, dm, mf, dv, kf, fx, dq, cw, nm, ox, px, rx, an, ui, sy, sh, tx, ct]

58. Fluorescent Antinuclear.mp. [mp=ab, hw, kw, ti, ot, tn, dm, mf, dv, kf, fx, dq, cw, nm, ox, px, rx, an, ui, sy, sh, tx, ct]

59. Anti-DNA antibod*.mp. [mp=ab, hw, kw, ti, ot, tn, dm, mf, dv, kf, fx, dq, cw, nm, ox, px, rx, an, ui, sy, sh, tx, ct]

60. Anti DNA Antibod*.mp. [mp=ab, hw, kw, ti, ot, tn, dm, mf, dv, kf, fx, dq, cw, nm, ox, px, rx, an, ui, sy, sh, tx, ct]

61. Anti Citrullinated Protein Antibod*.mp. [mp=ab, hw, kw, ti, ot, tn, dm, mf, dv, kf, fx, dq, cw, nm, ox, px, rx, an, ui, sy, sh, tx, ct]

62. Anti-Citrullinated Protein*.mp. [mp=ab, hw, kw, ti, ot, tn, dm, mf, dv, kf, fx, dq, cw, nm, ox, px, rx, an, ui, sy, sh, tx, ct]

63. Anti-Citrullinated.mp. [mp=ab, hw, kw, ti, ot, tn, dm, mf, dv, kf, fx, dq, cw, nm, ox, px, rx, an, ui, sy, sh, tx, ct]

64. Citrullinated Protein Antibod*.mp. [mp=ab, hw, kw, ti, ot, tn, dm, mf, dv, kf, fx, dq, cw, nm, ox, px, rx, an, ui, sy, sh, tx, ct]

65. Citrullinated Protein*.mp. [mp=ab, hw, kw, ti, ot, tn, dm, mf, dv, kf, fx, dq, cw, nm, ox, px, rx, an, ui, sy, sh, tx, ct]

66. Citrullinated.mp. [mp=ab, hw, kw, ti, ot, tn, dm, mf, dv, kf, fx, dq, cw, nm, ox, px, rx, an, ui, sy, sh, tx, ct]

67. Anti-Citrullinated Peptide Antibod*.mp. [mp=ab, hw, kw, ti, ot, tn, dm, mf, dv, kf, fx, dq, cw, nm, ox, px, rx, an, ui, sy, sh, tx, ct]

68. Anti Citrullinated Peptide Antibod*.mp. [mp=ab, hw, kw, ti, ot, tn, dm, mf, dv, kf, fx, dq, cw, nm, ox, px, rx, an, ui, sy, sh, tx, ct]

69. Anti-Citrullinated Peptide*.mp. [mp=ab, hw, kw, ti, ot, tn, dm, mf, dv, kf, fx, dq, cw, nm, ox, px, rx, an, ui, sy, sh, tx, ct]

70. Anti-Citrullinated.mp. [mp=ab, hw, kw, ti, ot, tn, dm, mf, dv, kf, fx, dq, cw, nm, ox, px, rx, an, ui, sy, sh, tx, ct]

71. Anti-Cyclic Citrullinated Protein Antibod*.mp. [mp=ab, hw, kw, ti, ot, tn, dm, mf, dv, kf, fx, dq, cw, nm, ox, px, rx, an, ui, sy, sh, tx, ct]

72. Anti Cyclic Citrullinated Protein Antibod*.mp. [mp=ab, hw, kw, ti, ot, tn, dm, mf, dv, kf, fx, dq, cw, nm, ox, px, rx, an, ui, sy, sh, tx, ct]

73. Anti-CCP.mp. [mp=ab, hw, kw, ti, ot, tn, dm, mf, dv, kf, fx, dq, cw, nm, ox, px, rx, an, ui, sy, sh, tx, ct]

74. Anti CCP.mp. [mp=ab, hw, kw, ti, ot, tn, dm, mf, dv, kf, fx, dq, cw, nm, ox, px, rx, an, ui, sy, sh, tx, ct]

75. Cyclic Citrullinated Peptide Antibod*.mp. [mp=ab, hw, kw, ti, ot, tn, dm, mf, dv, kf, fx, dq, cw, nm, ox, px, rx, an, ui, sy, sh, tx, ct]

76. Rheumatoid factor*.mp. [mp=ab, hw, kw, ti, ot, tn, dm, mf, dv, kf, fx, dq, cw, nm, ox, px, rx, an, ui, sy, sh, tx, ct]

77. Autoimmun*.mp. [mp=ab, hw, kw, ti, ot, tn, dm, mf, dv, kf, fx, dq, cw, nm, ox, px, rx, an, ui, sy, sh, tx, ct]

78. Autoimmun* disease*.mp. [mp=ab, hw, kw, ti, ot, tn, dm, mf, dv, kf, fx, dq, cw, nm, ox, px, rx, an, ui, sy, sh, tx, ct]

79. 29 or 30 or 31 or 32 or 33 or 34 or 35 or 36 or 37 or 38 or 39 or 40 or 41 or 42 or 43 or 44 or 45 or 46 or 47 or 48 or 49 or 50 or 51 or 52 or 53 or 54 or 55 or 56 or 57 or 58 or 59 or 60 or 61 or 62 or 63 or 64 or 65 or 66 or 67 or 68 or 69 or 70 or 71 or 72 or 73 or 74 or 75 or 76 or 77 or 78

80. 26 and 28 and 79

81. limit 80 to yr="2019 -Current"

Table S1. Characteristics of included studies.

Study	Population size (n)	Country	Experimental group	Control group	Autoantibody and methodology	Patients characteristics	Outcomes
Abers et al. (28)	218	Italy	26 anti-IFN hospitalized COVID-19 patients (RT-PCR; >18 years)	192 hospitalized COVID-19 patients without anti-IFN	Anti-IFN alfa, IFN-omega, IFN-beta (multiplex particle-based flow cytometry, ELISA, LIPS, functional activity blocking); performed at admission	135 critical, 44 severe, 39 mild/moderate; median age: 62.9 years; male sex: 74.4% (p 0.03); severity defined by Chinese National Health Commission (CNHC)	Intensive care; invasive mechanical ventilation; bacterial infections; mortality (10 weeks); severity
Gonen et al. (29)	74	Turkey	49 hospitalized COVID-19 patients (RT-PCR; >18 years)	25 healthy controls	Antipituitary and antihypothalamica antibodies (indirect immunofluorescence)	26.5% mild, 36.7% moderate, 36.7% severe; no age and sex differences; severity by CNHC	Severity
Guasp et al. (30)	130	Spain	60 hospitalized neuro-COVID-19 patients (RT-PCR; >18 years)	70 healthy controls	Diverse anti-neuronal antibodies (immunohistochemistry, indirect immunofluorescense, immunoblot)	Mean age: 66; male sex: 60%	Neuro-COVID-19
Karahan et al. (31)	59	Turkey	31 critical hospitalized COVID-19 patients	28 severe non-COVID 19 patients	Anti-cardiolipin IgG/IgM (ELISA), anti-beta 2-glycoprotein I IgG/IgM/IgA (ELISA), Lupus anticoagulant (fully automated coagulometer device)	Mean age: 60 years; male sex: 60%	Severity; thrombotic events
L'Huillier et al. (32)	193	Switzerland	193 COVID-19 patients (RT-PCR; >18 years)	0	Anti-apoliporotein A1 IgG (ELISA); performed until 6 weeks after diagnosis	Median age: 40.6 years; male sex: 30%; 97% outpatients	Post-COVID-19 syndrome
Lui et al. (33)	122	China	122 hospitalized COVID-19 patients (RT-PCR; >18 years) without thyroid diseases	0	Anti-thyroid peroxidase, anti-thyroglobulin, anti-thyroid stimulating hormone receptor (ELISA), performed at admission and 3 months apart	Mean age: 58 years; male sex: 49%; severe COVID-19: 3%	Severity
Najim et al (34)	60	Qatar	60 critical COVID-19 patients (RT-PCR; >18 years)		Anticardiolipin IgG/IgM, anti-beta 2-glycoprotein I IgG/IgM, Lupus anticoagulant (coagulometer), performed until 72 hours after admission	Mean age: 52.8 years; male sex: 95%	Mortality (60 days); thrombosis
Pagano et al. (35)	126	Switzerland	126 critical COVID-19 patients (RT-PCR; > 18 years)	1326 patients	Anti-apolipoprotein A1 IgG (ELISA)	Median age: 63.5 years; male sex: 77,8%	Mortality (28 days); Severity (SAPS II, Apache II, SOFA); mechanical ventilation
Pascolini et al. (9)	58	Italy	33 hospitalized COVID-19 patients (interstitial pneumonia: 94%; RT-PCR; >18 years)	25-age matched controls with fever or non-COVID-19 pneumonia	ANA, ANCA, ENA, anticardiolipin IgG/IgM, anti-beta2-glycoprotein I IgG/IgM, anti-proteinase 3, anti-myeloperoxidase (indirect immunofluorescense, immunoblot, FEIA)	Median age: 70 years; male sex: 51.6%; deaths related to COVID-19: 21%; critical: 27.2%	Severity; mortality
Peker et al. (36)	100	Turkey	50 hospitalized COVID-19 patients (RT-PCR; >18 years)	50 pre-pandemic healthy controls	ANA, anti-DNA, ANCA (indirect immunofluorescense), anti-CCP (chemiluminescense), ENA (Euroline ANA-profile 1 kit) performed 7 days (median) after diagnosis	Male sex: 50%; mean age: 58.8 years	Mortality (acute phase); severity
Petrikov et al. (37)	127	Russia	70 critical COVID-19 patients (RT-PCR; >18 years)	Serum for 57 prior mild COVID-19 patients	Anti-IFN alpha (ELISA) performed 8-50 days after onset of illness	Median age: 68 years; male sex: 52%; NEWS2 scale for severity	Mortality (acute phase); mechanical ventilation
Raadsen et al. (38)	431	Netherlands	282 COVID-19 patients (RT-PCR; >18 years)	103 pre-pandemic healthy controls + 46 critical non-COVID-	Anti-IFN (ELISA + neutralization assay) performed 51 days after symptoms (mean) and during follow-up (1 week apart)	Male sex: 81.2%; mean age: 57 years; severity by WHO, mild: 100/282; moderate: 43/282; severe: 97/282; fatal 38/282	Severity; survival (60 days)

				19 patients			
Rodriguez-Perez et al. (39)	142	Spain	119 COVID-19 patients (RT-PCR; >18 years)	23 healthy controls	Anti-ACE2 and anti-angiotensin I (ELISA)	Severity by WHO, mild 31/119; moderate 68/119; severe 20/119; mean age: 56.5 years; male sex: 48.3%	Severity
Sacchi et al. (40)	80	Italy	40 hospitalized COVID-19 patients (RT-PCR; >18 years)	40 healthy controls	Anticardiolipin IgG/IgM, anti-beta2-glicoprotein I IgG/IgM (chemiluminescense), ASCA IgG/IgM, anti-MPO, anti-Pr3, anti-RNP, anti-Ro, anti-La, anti-centromere, anti-fibrillarin, anti-Mi2, anti-PM/SCL, anti-SCL 70, anti-Jo1, anti-Sm, anti-DNA (ELISA), ANCA and ANA (indirect immunofluorescense); Performed at hospital admission	Mean age: 66.8 years; mortality: 27.5%	Mortality (acute phase)
Seeble et al. (41)	96	Germany	96 COVID-19 patients (RT-PCR; >18 years)	0	ANA (indirect immunofluorescence) performed in acute phase (until 16 days post symptoms) and follow-up visits	32.3% hospitalized; male sex: 57.4%; median age: 65 years	Post-COVID-19 syndrome; Severity
Serrano et al. (42)	833	Spain	474 severe hospitalized COVID-19 patients (RT-PCR; >18 years)	359 healthy controls	Anticardiolipin IgG/IgM, anti-beta2-glycoprotein I IgG/IgM/IgA, anti-phosphatidylserine/anti-thrombin IgG/IgM (ELISA); performed until 24 hours post diagnosis	Median age: 65 years; male sex: 62.8%	Mortality (30 days); survival; thrombosis; ventilatory failure;
Su et al. (43)	666	United States	209 COVID-19 patients (RT-PCR; >18 years)	457 healthy controls	Anti-IFN alpha 2, anti-Ro, anti-La, anti-RNP, anti-Jo1, anti-P1 (ELISA)	Mean age: 53 years; Male sex: 42%; severity by WHO; hospital admission: 71% in INCOV cohort; 10% in HAARVI cohort	Post-COVID-19 syndrome
Taeschler et al. (44)	216	Sweden, England, Switzerland	175 COVID-19 patients (RT-PCR; >18 years)	41 controls	ANCA, ANA (indirect immunofluorescense), Anti-MPO and anti-pr3 (ELISA)	Severity by WHO; severe: 37.7%; median age: 50.5 years; male sex: 55.6%	Severity

ANA: anti-nuclear antibodies; ANCA: antineutrophil cytoplasmic antibodies; ENA: extractable nuclear antigens; CCP: citrulinated cyclic protein; WHO: World Health Organization; ACE2: angiotensin converting enzyme 2.
